# CD11c-specific bio-nanocapsule enhances vaccine immunogenicity by targeting immune cells

**DOI:** 10.1186/s12951-018-0386-6

**Published:** 2018-08-04

**Authors:** Hidenori Matsuo, Masaharu Somiya, Masumi Iijima, Takeshi Arakawa, Shun’ichi Kuroda

**Affiliations:** 1 Graduate School of Bioagricultural Sciences, Nagoya University, Nagoya, 464-8601 Japan; 2 Department of Biomolecular Science and Reaction, The Institute of Scientific and Industrial Research, Osaka University, Mihogaoka 8-1, Ibaraki, Osaka 567-0047 Japan; 3 Department of Nutritional Science and Food Safety, Faculty of Applied Bioscience, Tokyo University of Agriculture, Tokyo, 156-8502 Japan; 4 COMB, Tropical Biosphere Research Center, University of the Ryukyus, Nishihara, Okinawa 903-0213 Japan; 5 Graduate School of Medicine, University of the Ryukyus, Nishihara, Okinawa 903-0215 Japan

**Keywords:** Bio-nanocapsule, Hepatitis B virus, Vaccine, Dendritic cell, In vivo targeting, Protective immunity

## Abstract

**Background:**

Various nanocarriers have been used to deliver subunit vaccines specifically to dendritic cells (DCs) for the improvement of immunogenicity. However, due to their insufficient DC priming ability, these vaccines could not elicit effective innate immunity. We have recently developed a DC-targeting bio-nanocapsule (BNC) by displaying anti-CD11c IgGs via protein A-derived IgG Fc-binding Z domain on the hepatitis B virus envelope L protein particles (α-DC-ZZ-BNC).

**Results:**

After the chemical modification with antigens (Ags), the α-DC-ZZ-BNC-Ag complex could deliver Ags to DCs efficiently, leading to effective DC maturation and efficient endosomal escape of Ags, followed by Ag-specific T cell responses and IgG productions. Moreover, the α-DC-ZZ-BNC modified with Japanese encephalitis virus (JEV) envelope-derived D3 Ags could confer protection against 50-fold lethal dose of JEV injection on mice.

**Conclusion:**

The α-DC-ZZ-BNC-Ag platform was shown to induce humoral and cellular immunities effectively without any adjuvant.

**Electronic supplementary material:**

The online version of this article (10.1186/s12951-018-0386-6) contains supplementary material, which is available to authorized users.

## Background

Subunit vaccines have been utilized for avoiding the latently side-effects of conventional vaccines (e.g., live, live-attenuated, and inactivated vaccines), whereas low immunogenicity often observed in subunit vaccines has remained to be solved. Drug delivery system (DDS)-based subunit vaccines, delivering antigens (Ags) to Ag-presenting cells (APCs), have therefore become promising for enhancing the immunogenicity of conventional subunit vaccines. Especially, since dendritic cells (DCs) play a pivotal role in adaptive immunity, various nanocarriers have been targeted to DCs by displaying molecules recognizing DC-specific receptors (integrin [1], C-type lectin [2], and Fc receptors [3]), and thereby these nanocarriers have shown to enhance the immunogenicity of loaded Ags in vivo [4, 5]. Meanwhile, it has been known that priming of innate immunity is necessary for eliciting following adaptive immunity [6]. Because these nanocarriers are less recognized with pattern recognition receptors (PRRs) including Toll-like receptors, they require additional adjuvants consisting of PRR ligands [7], while it is still necessary to elucidate the DC priming mechanism on the molecular basis for the assurance of their safety. Furthermore, the intracellular kinetics of delivered Ags in DCs should be controlled for eliciting effective humoral and cellular immunities. Incorporated Ags in endosomes are processed to small peptides by proteinases for presenting to naïve CD4^+^ T cells via major histocompatibility complex (MHC) class II, leading to the initiation of helper T cell (Th cell)-dependent immunity (i.e., humoral immunity). On the other hands, a part of Ags are translocated from endosome to cytoplasm, processed to small peptides by proteasomes, and then transferred to endoplasmic reticulum for presenting to naïve CD8^+^ T cells via MHC class I, leading to the initiation of cytotoxic T lymphocyte (CTL)-dependent immunity (i.e., cellular immunity) [8]. For example, the Ag-loaded liposomes (LPs) modified with pH-sensitive fusogenic polymer could release Ags efficiently to cytoplasm by endosomal membrane fusion under acidic condition in late-endosomes, which elicit more effective CTL activity rather than unmodified LPs [9]. Thus, DC-targeting nanocarriers should control intracellular kinetics of Ags in DCs to induce the most effective immune responses against each pathogen. Generally, prophylactic vaccine is based on humoral immunity, contributing to neutralizing pathogens, facilitating phagocytosis, and complement fixation. In contrast, therapeutic vaccine is mainly based on cellular immunity, eliminating pathogens and infected cells [10, 11].

Bio-nanocapsule (BNC) is an approximately 50-nm hollow nanoparticle consisting of hepatitis B virus (HBV) surface Ags L proteins, which could be synthesized in recombinant yeast cells [12]. Since N-terminal region of L protein harbors both HBV-derived human liver-specific recognition domain and membrane fusogenic domain concurrently [13–16], BNCs could function as a nanocarrier specific to human hepatic cells with comparable infectivity of HBV [17, 18]. Followed by the fusion with LPs containing payloads, BNC-LP complex could deliver them to human hepatic cells specifically [19, 20]. Moreover, our group established mutated BNC (ZZ-BNC) by replacing the human liver-specific recognition domain with a tandem form of IgG Fc-binding Z domain from *Staphylococcus aureus* protein A to display IgGs outwardly in an oriented immobilization manner [21]. We have identified that anti-CD11c IgGs (clone N418)-displaying ZZ-BNC (α-DC-ZZ-BNC) could accumulate into splenic DCs in mice through intravenous (IV) injection [22]. Next, the α-DC-ZZ-BNC complex was fused with Ag-loaded cationic LPs (LP-Ag), and the α-DC-ZZ-BNC-LP-Ag complex injected intravenously could efficiently induce Ag-specific IgG production rather than Ag alone. Thus, α-DC-ZZ-BNC has advantages in effective elicitation of Ag-specific immunity by DC-specific Ag delivery. Although vaccines have been usually injected through subcutaneous (SC) and intramuscular (IM) routes at worldwide clinical sites, the SC-injected α-DC-ZZ-BNC-LP-Ag complex was found less immunogenic unexpectedly. It was postulated that the LP-derived positive charge might disturb the movement of the complex from injection site to DCs (migratory DCs, lymph organ-resident DCs). On the other hand, we have recently developed a vaccine platform consisting of Ag-crosslinked ZZ-BNC [23]. When ZZ-BNC was chemically conjugated with Japanese encephalitis virus (JEV)-derived D3 Ag, the SC-injected ZZ-BNC-D3 complex could induce JEV-specific neutralizing IgG production more effectively than D3 alone in mice. These situations have led us to examine if the α-DC-ZZ-BNC-Ag complex induces more effective immunity than ZZ-BNC-Ag even through local injections.

In this study, we have formulated the α-DC-ZZ-BNC-Ag complex by using model Ag ovalbumin (OVA), and demonstrated that the complex could deliver Ags to splenic DCs ex vivo. The complex was found to induce DC maturation without any adjuvant, followed by efficient endosomal escape of Ags, CTL proliferation, and Th1/Th2 immune responses. Furthermore, for demonstrating that the complex is more effective platform for prophylactic vaccines than conventional subunit vaccines, we examined the protective efficacy of α-DC-ZZ-BNC-D3 complex against Japanese encephalitis virus (JEV) infection in mice.

## Results

### Preparation of α-DC-ZZ-BNC-OVA complex

ZZ-BNC was conjugated with anti-CD11c IgGs and model Ags OVA. Based on the densitometric intensities of stained bands in SDS-polyacrylamide gel electrophoresis analysis, single α-DC-ZZ-BNC-OVA complex was estimated to contain approximately 128 molecules of OVA and 8 molecules of α-CD11c IgG. Therefore, the weight ratio of OVA to ZZ-BNC in the formulation was estimated as approximately 1:0.8. As shown in Table 1, each complex is less than 100 nm of diameter and negatively charged (~ − 30 mV), which were considered suitable for in vivo Ag-delivery to DCs in the lymph organs closest to injection sites [24].

**Table 1:** Particle properties of α-DC-ZZ-BNC-OVA analyzed by a dynamic light scattering

Samples	Z-average (nm)	PDI	ζ-potential (mV)
BNC-OVA	91.5 ± 1.1	0.236	− 33.3 ± 4.1
ZZ-BNC-OVA	68.0 ± 8.2	0.236	− 30.2 ± 1.7
α-DC-ZZ-BNC-OVA	68.2 ± 9.7	0.215	− 27.9 ± 4.0
IgG-ZZ-BNC-OVA	80.9 ± 20	0.222	− 26.3 ± 6.8

N = 3, values are indicated as mean ± SD

### Cellular uptake of α-DC-ZZ-BNC-OVA by splenic DCs

**Fig. 1:**
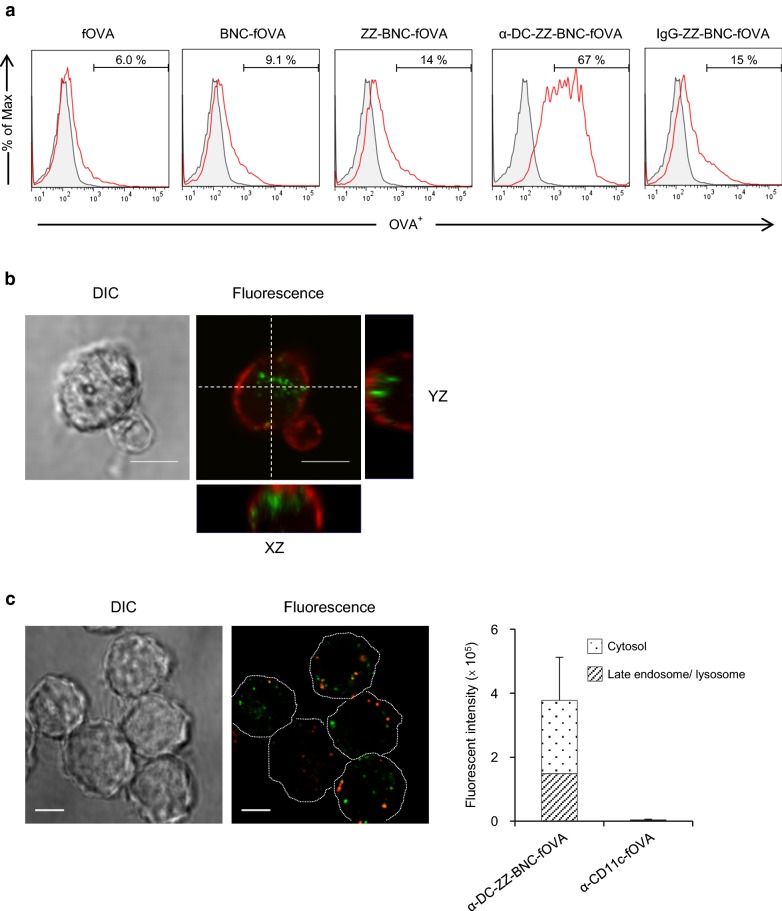
Cellular uptake of Ag-crosslinked α-DC-ZZ-BNC. **a** Isolated splenic DCs were incubated with each complex. The values were indicated percentages of fOVA^+^ cells in DCs. **b** The isolated DCs were treated with α-DC-ZZ-BNC-fOVA (green), and stained with wheat germ agglutinin (Red). Scale bars, 5 μm. **c** The DC2.4 cells were incubated with α-DC-ZZ-BNC-fOVA (green) and stained with LysoTracker Red (Red). Scale bars, 5 μm. Fluorescence intensity of fOVA in the DC2.4 cells was analyzed with an ImageJ software (N = 7, mean ± SEM)

When fOVA-crosslinked α-DC-ZZ-BNC (α-DC-ZZ-BNC-fOVA) was incubated with splenic DCs, fOVA was accumulated to 67% of DCs, while fOVA alone, BNC-fOVA (without ZZ domain), ZZ-BNC-fOVA, and IgG-ZZ-BNC-fOVA were estimated to 6.0%, 9.1%, 14%, and 15% of DCs, respectively (Fig. 1a). This result indicated that the conjugation with anti-CD11c IgGs could confer DC-targeting capability on ZZ-BNC-OVA. Furthermore, it was suggested that ZZ domains help the accumulation to DCs by interacting with Ig molecules on the cell surface. As shown in Fig. 1b, fOVA was localized inside of DCs, indicating that α-DC-ZZ-BNC-fOVA could deliver fOVA to the intracellular fraction of DCs efficiently. Moreover, α-DC-ZZ-BNC-fOVA could deliver approximately 61% of fOVA into the cytosol compartments of DC2.4 cells (Fig. 1c).

### In vivo DC-targeting through local injections

**Fig. 2:**
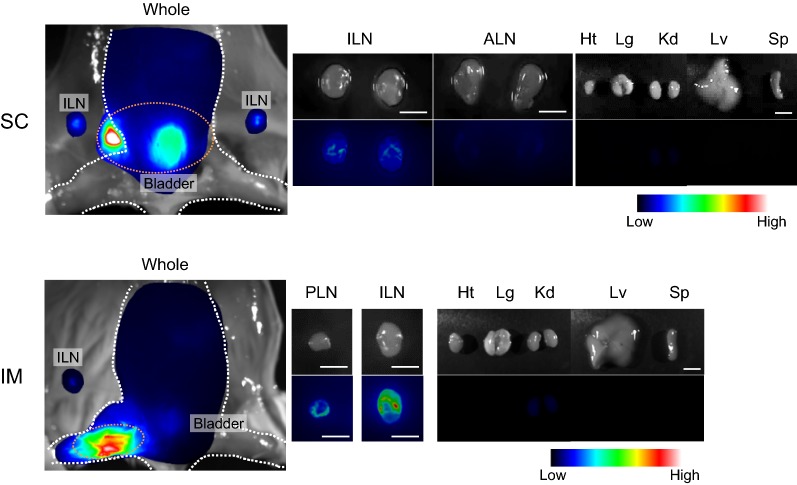
In vivo distribution of α-DC-ZZ-BNC injected through SC and IM routes. CF750-labeled α-DC-ZZ-BNC was injected to mice through SC and IM. After 12 h, mice were sacrificed, and tissues [heart, Ht; lung, Lg; kidney, Kd; liver, Lv; spleen, Sp; LNs (inguinal, ILN; popliteal, PLN; axillary, ALN)] were isolated. Scale bar in LN images, 3 mm; in organ images, 10 mm

In this study, we examined whether α-DC-ZZ-BNC could be used for the targeting DCs through SC and IM routes (Fig. 2). When mice were administrated with α-DC-ZZ-BNC through SC or IM routes, substantial amount of fluorescence was observed in LNs closest to injection sites (ILNs for SC, ILN and PLN for IM) and small amount of fluorescence in kidney, while large amount of fluorescence was observed at injection sites and bladder. The accumulation of α-DC-ZZ-BNC complex in LNs strongly suggested that the complex was captured by APCs at either injection sites or LNs.

**Fig. 3:**
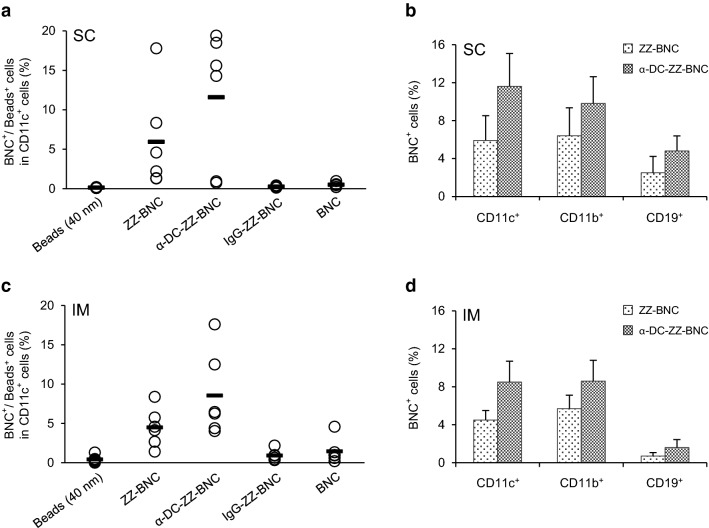
In vivo accumulation of α-DC-ZZ-BNC injected through local routes to APCs in LNs. LN cells were isolated and stained with α-CD11c-FITC for evaluating the accumulation of each particle to DCs in ILN (**a**) or PLN (**c**). Accumulation of α-DC-ZZ-BNC to CD11c^+^ cells, CD11b^+^ cells, and CD19^+^ cells in ILN (**b**) or PLN (**d**) was analyzed (N = 6, mean ± SEM)

For deciphering target cells of α-DC-ZZ-BNC complex in each LN, the APC subsets of LNs was analyzed by flow cytometer. As shown in Fig. 3a, α-DC-ZZ-BNC complex could target approximately 12% of CD11c^+^ cells (i.e., DCs) in ILNs more efficiently than ZZ-BNC, IgG-ZZ-BNC, BNC, and 40-nm beads after SC injection. When comparing with ZZ-BNC, the degree of DC targeting could be enhanced by anti-CD11c IgGs to some extent. Moreover, α-DC-ZZ-BNC and ZZ-BNC could accumulate to CD11b^+^ cells (including macrophages and myeloid DCs) at comparable level, and CD19^+^ cells (B cells) to less extent (Fig. 3b). On the other hand, α-DC-ZZ-BNC complex injected IM could target approximately 9% of CD11c^+^ cells in PLN, which was higher than ZZ-BNC, IgG-ZZ-BNC, BNCs, and 40-nm fluorescent beads (Fig. 3c). As for the accumulation of α-DC-ZZ-BNC and ZZ-BNC to CD11b^+^ cells (e.g., macrophages, myeloid DCs) and CD19^+^ cells (B cells), any significant difference could not be found between SC and IM injections (Fig. 3b, d). The accumulation of these complexes to CD11b^+^ cells might be caused by high mannose-type sugar chains of ZZ-BNC that binds to mannose receptors [8]. In addition, the accumulation of these complexes to CD19^+^ cells might be mediated by the interaction of ZZ domains with Ig molecules [8]. These results strongly suggested that the α-DC-ZZ-BNC complex could efficiently target wide variety of APCs.

### Induction of DC maturation with α-DC-ZZ-BNC-OVA

**Fig. 4:**
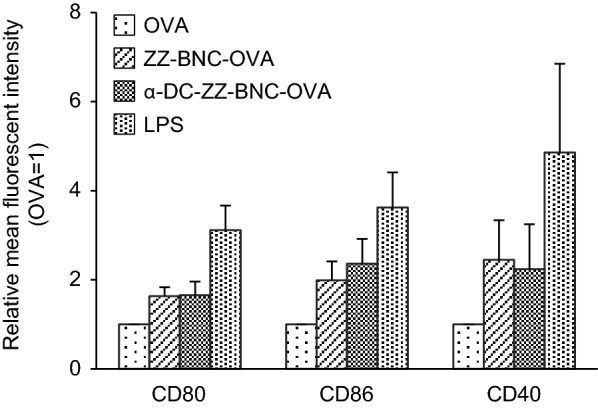
Induction of DC maturation with α-DC-ZZ-BNC-OVA. Splenic DCs were incubated with OVA, ZZ-BNC-OVA, and α-DC-ZZ-BNC-OVA. As positive control, LPS was added to the culture (N = 3, mean ± SEM)

CD80 and CD86, play an important role in DC maturation, followed by Ag-presentation for providing stimulatory signals to naïve T cells. CD40 mediates subsequent DC maturation by the interaction with CD40 ligands [25]. It was known that either mannose-type sugar chains or anti-CD11c antibodies could induce DC maturation [26, 27]. Since α-DC-ZZ-BNC has both high mannose-type sugar chains and α-CD11c IgGs, we examined if the α-DC-ZZ-BNC-OVA complex could elicit DC maturation without any adjuvant. Isolated splenic DCs were incubated with α-DC-ZZ-BNC-OVA, and then the expression levels of CD80, CD86, and CD40 were analyzed. The expression level of CD80 was elevated with ZZ-BNC-OVA and α-DC-ZZ-BNC-OVA by 1.6-fold and 1.7-fold (OVA alone as control), respectively (Fig. 4). The expression levels of CD86 and CD40 were also elevated with ZZ-BNC-OVA and α-DC-ZZ-BNC-OVA to similar extent. It was therefore revealed that both α-DC-ZZ-BNC-OVA and ZZ-BNC-OVA could elicit effective ex vivo DC maturation without any adjuvant.

### Induction of OVA-specific CTL proliferation with α-DC-ZZ-BNC-OVA

**Fig. 5:**
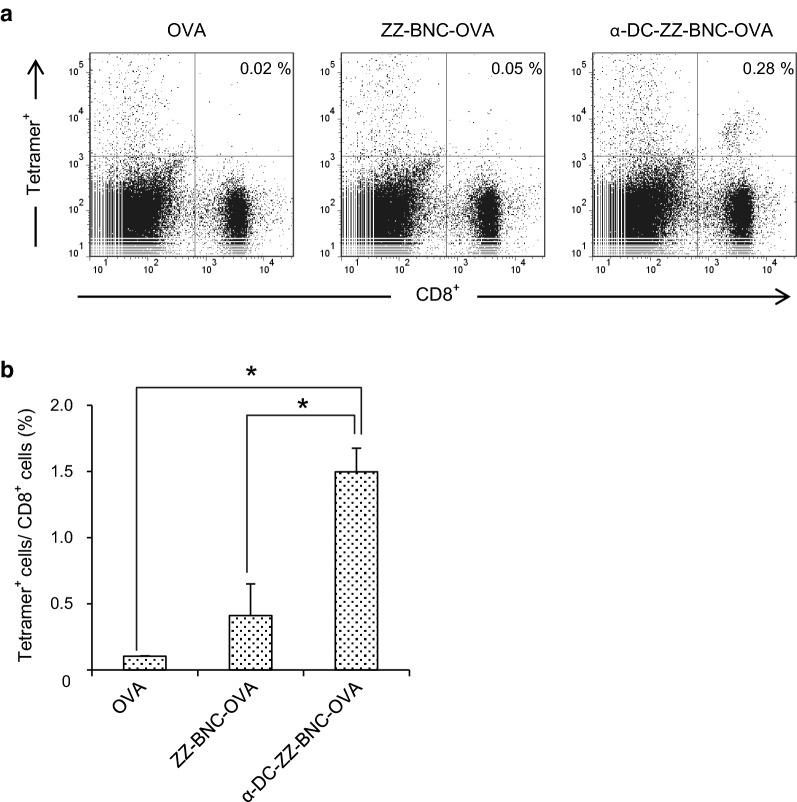
Proliferation of OVA-specific CTL by immunization with α-DC-ZZ-BNC-OVA. **a** After 7 days from subcutaneous injection, C57BL/6 mice were sacrificed, and the splenocytes were isolated and stained with APC-labeled H-2kb OVA tetramer and FITC-labeled α-CD8 IgG. **b** Statistic analysis of the CTL proliferation (N = 3, mean ± SEM). *p < 0.01

The cytotoxic T cell responses in OVA-immunized mice were evaluated using H-2k^b^ OVA tetramer-SIINFEKL (fluorescence probe for OVA-specific CTL) and anti-CD8-FITC (Fig. 5a). Comparing with OVA alone, the ZZ-BNC-OVA and α-DC-ZZ-BNC-OVA could enhance the proliferation of OVA-specific CD8^+^ T lymphocytes moderately and efficiently, respectively (Fig. 5b).

### Evaluation of Th1- and Th2-related immune responses induced with α-DC-ZZ-BNC-OVA

**Fig. 6:**
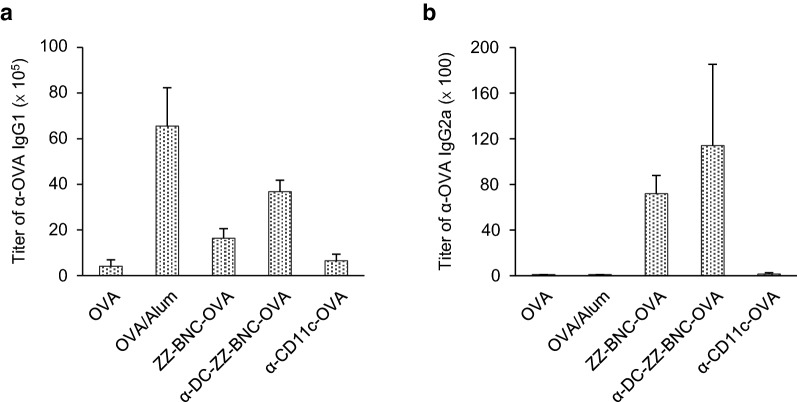
Induction of OVA-specific IgGs production with α-DC-ZZ-BNC-OVA. Mice received subcutaneous injection of α-DC-ZZ-BNC-OVA. Sera were collected from the immunized mice, and then titers of OVA-specific IgG1 (**a**) and IgG2a (**b**) in sera were determined by ELISA (N = 6, mean ± SEM)

Mice were immunized with various forms of OVA and anti-OVA IgG1 and IgG2a in sera were measured. As for the Th2-dependent immunity, both α-DC-ZZ-BNC-OVA and ZZ-BNC-OVA induced anti-OVA IgG1 efficiently (Fig. 6a). Both IgG-ZZ-BNC-OVA and α-CD11c-OVA induced lower level of anti-OVA IgG1. As for the Th1-dependent immunity, while OVA/Alum (positive control for Th2-dependent immunity [7]) and α-CD11c-OVA were less immunogenic, all BNC-containing vaccines showed about 100-fold higher level of α-OVA IgG2a induction than other vaccines (Fig. 6b). These results suggested that BNC-containing vaccines could induce Th1 and Th2 immunities effectively. However, there was no significant difference among BNC-containing vaccines, indicating that anti-CD11c IgG is necessary for the production of IgGs. The components of ZZ-BNC and clustered Ags may contribute to the production of IgGs through sufficient DC maturation.

### Protective immunity induced with α-DC-ZZ-BNC-based vaccine

**Fig. 7:**
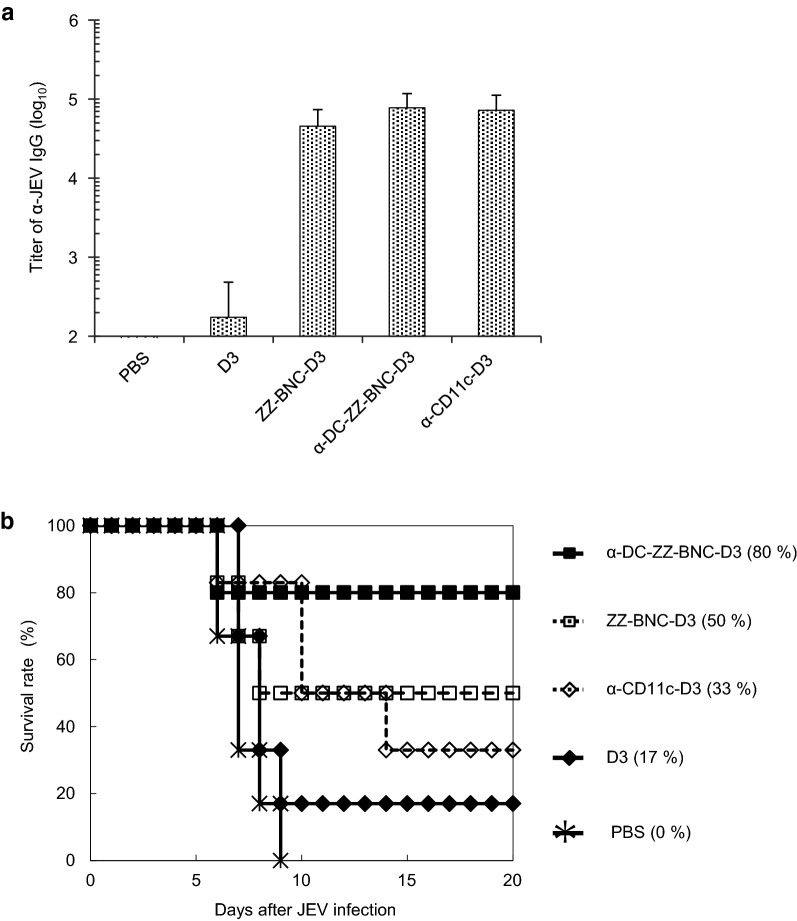
Induction of protective immunity against JEV infection with α-DC-ZZ-BNC-D3. Mice were received subcutaneous injection of α-DC-ZZ-BNC-D3 at week 0, 2, and 4. **a** Titers of JEV-specific total IgG in sera were determined by using ELISA (N = 6, mean ± SEM). **b** The mice immunized with α-DC-ZZ-BNC-D3 were challenged intraperitoneally with 50-times dose of 50% lethal dose of the JEV JaGAr01 strain followed by intracerebral inoculation with PBS

We evaluate the effect of DC targeting by α-CD11c IgG on the protective efficacy against JEV challenge. Bacterially expressed JEV-derived D3 was conjugated with α-DC-ZZ-BNC to form α-DC-ZZ-BNC-D3. The particle properties of the complex were similar to those of α-DC-ZZ-BNC-OVA (Table 2). Mice were immunized with α-DC-ZZ-BNC-D3, α-CD11c-D3 (BNC-less control), ZZ-BNC-D3 (α-CD11c IgG-less control), and D3 alone through SC injection. As shown in Fig. 7a, α-DC-ZZ-BNC-D3 could induce α-JEV total IgG production more efficiently than D3 alone, but there was no significant difference among α-DC-ZZ-BNC-D3, ZZ-BNC-D3, and α-CD11c-D3.

**Table 2:** Particle properties of α-DC-ZZ-BNC-D3 analyzed by a dynamic light scattering

Samples	Z-average (nm)	PDI	ζ-potential (mV)
ZZ-BNC-D3	70.2 ± 10	0.324	− 23.7 ± 7.0
α-DC-ZZ-BNC-D3	74.6 ± 14	0.341	− 24.3 ± 7.2

N = 3, mean ± SD

Immunized mice were inoculated with JEV intraperitoneally, and then survival rates were recorded for 20 days (Fig. 7b). Immunization with α-DC-ZZ-BNC-D3 could protect 80% of the immunized mice from JEV challenge, while survival rates of the mice immunized with ZZ-BNC-D3, α-CD11c-D3, and D3 alone were 50%, 33%, and 17%, respectively. Thus, α-DC-ZZ-BNC-D3 could induce most effective protective immunity among vaccines used. Although the titers of α-JEV IgGs elicited by α-DC-ZZ-BNC-D3, ZZ-BNC-D3, and α-CD11c-D3 were comparable (Fig. 7a), the DC targeting driven by α-CD11c IgG successfully contributed to the survival of JEV-challenged mice. This result suggested that the DC targeting could enhance cellular immunity rather than humoral immunity.

## Discussion

DC-specific Ag delivery has been considered as a promising strategy for facilitating efficient recognition, processing, and presentation of Ags by DCs, leading to enhance the Ag-specific immunity. Generally, for elicitation of adaptive immunities, vaccines should induce effective innate immunity by providing stimulatory signals to APC via PRRs (including Toll-like receptors) [6]. Adjuvants can elicit innate immunity by interacting with PRRs to stimulate the secretion of cytokines [7]. However, either non-specific or over elicitation of innate immunity by adjuvants should be avoided for the guarantee of their safety, because wide variety of host cells harbors PRRs. These situations have led us to deliver Ags in a DC-specific manner by using DC-targeting nanocarriers. Previously, it was revealed that α-DC-ZZ-BNC could accumulate into splenic DCs through IV injection and the α-DC-ZZ-BNC-LP-Ag complex could elicit Ag-specific IgG production efficiently [22]. For optimizing the particle properties (e.g., charge, size) of α-DC-ZZ-BNC-based vaccines, the Ag incorporation method was changed from LP fusion to chemical crosslinking. When fOVA was used as model Ag, the α-DC-ZZ-BNC-fOVA could deliver fOVA into DCs ex vivo efficiently. Importantly, the fOVA delivered with α-DC-ZZ-BNC-fOVA was localized at the intracellular fraction of DCs (Fig. 1a, b). CD11c molecule forms heterodimeric complement receptor 4 (CR4) with CD18 molecule, involved in the incorporation of pathogens by DCs [28]. Cellular uptake of fOVA by CR4 might be enhanced by targeting CD11c molecules with α-DC-ZZ-BNC. Additionally, the large amount of fOVA delivered with α-DC-ZZ-BNC-fOVA was translocated into cytosol compartment in DCs (Fig. 1c), suggesting that the Ag translocation could facilitate cellular immunity depending on MHC class I (i.e., cross-presentation). Membrane fusogenic activity from HBV retained in N-terminal of L and ZZ-L protein could mediate endosomal escape of BNCs into cytosol compartments [14]. Since cytosolic delivery of Ags could enhance MHC class I-dependent Ag presentation leading to CTL-dependent cellular immunity, the vaccine platform in this study might be suitable for inducing effective cellular immunity.

Followed by the formulation of α-DC-ZZ-BNC, the complex was administrated via local injections (SC and IM). In vivo imaging analysis indicated the accumulation of α-DC-ZZ-BNC to the LNs closest to the injection sites (Fig. 2). Anti (α)-DC-ZZ-BNC injected either SC or IM accumulated to approximately 10% of DCs in the LNs (Fig. 3a, c). Interestingly, both α-DC-ZZ-BNC and ZZ-BNC could accumulate not only to DCs but also to CD11b^+^ cells and CD19^+^ cells, corroborating the pleiotropic APC-targeting function of ZZ-BNC is sustained by the interactions of its components with APC surface receptors (i.e., the interaction of ZZ domains and mannose-type sugar chains with Ig molecules and C-type lectin receptors (CLR), respectively). Moreover, α-DC-ZZ-BNC is the most effective APC-targeting nanocarrier driven by synergistic action of α-CD11c IgGs and ZZ-BNC components.

Although conjugation of α-CD11c IgG contributed to Ag-dependent CTL proliferation (Fig. 5) and induction of protective immunity (Fig. 7b), no contribution was observed in induction of DC maturation (Fig. 4), Ag-dependent IgG production (Fig. 6), and IgG class switch (Fig. 6). These results demonstrated that α-DC-ZZ-BNC-Ag rather than ZZ-BNC-Ag could elicit T cell-dependent immune responses effectively. Taking the advantage of α-CD11c IgG in in vivo DC targeting into considerations, α-DC-ZZ-BNC-Ag could presumably induce in vivo DC maturation more robustly than ZZ-BNC-Ag. Since the incorporation of Ag with α-CD11c IgG could induce effective DC maturation through the interaction with CR4 in a Toll-like receptor-independent manner [29], the α-CD11c IgG moiety of α-DC-ZZ-BNC-Ag could also induce in vivo DC maturation, followed by the induction of subsequent humoral and cellular immunities.

While α-JEV IgG titer of mice immunized with α-DC-ZZ-BNC-D3 was comparable to that with ZZ-BNC-D3, it was clearly demonstrated that α-DC-ZZ-BNC-OVA rather than ZZ-BNC-OVA could induce antigen-specific CTLs efficiently (Fig. 5). This result suggested that α-DC-ZZ-BNC could elicit stronger cellular immunity than ZZ-BNC. Previously, it was shown that cellular immunity contributes to the survival of mice challenged with JEV even without significant induction of α-JEV antibodies [30]. Therefore, cellular immunity may play a key role when the humoral immunity is not sufficiently induced for protecting mice completely from lethal JEV infection. Thus, it was concluded that α-DC-ZZ-BNC could induce both cellular and humoral immunity efficiently, and protect mice from lethal JEV infection through the cellular immunity.

As discussed previously, ZZ-BNC harbors three important functions for inducing immunological responses (i.e., ZZ domains, mannose-type sugar chains, Ag clustering). It was demonstrated that ZZ-BNC by itself functions as an APC-specific DDS nanocarrier. ZZ domains might interact with Ig molecules on B cells, leading to Ag presentation [31]. Mannose-type sugar chains might interact with CLRs (e.g., DC-SIGN, DNGR-1), eliciting innate immunity [32]. Especially, while α-DC-ZZ-BNC is the most potent APC-specific DDS nanocarrier in this study, α-CD11c IgG is operative only in mice. The ZZ-BNC is a more versatile DDS nanocarrier for wide variety of animals than α-DC-ZZ-BNC.

Comparing α-CD11c-D3 with ZZ-BNC-D3, the protective immunity against JEV challenge was more successful for ZZ-BNC-D3 (Fig. 7b), while there was little difference in D3-dependent IgG production (Fig. 7a). It was therefore postulated that ZZ-BNC-D3 could induce D3-specific CTL proliferation more effectively than α-CD11c-D3. When OVA was used instead of D3, both vaccines could induce sufficient α-OVA IgG1 without any adjuvant, which is comparable level of OVA with Alum (Fig. 6a). Meanwhile, α-CD11c-OVA could induce less α-OVA IgG2a than ZZ-BNC-OVA (Fig. 6b). Collectively, as described above, the ZZ-BNC components would stimulate PRRs and consequently induce robust Th1 and Th2 immunities.

## Conclusions

Both α-DC-ZZ-BNC-Ag and ZZ-BNC-Ag are revealed as a promising APC-targeting nanocarrier for forthcoming vaccines. They could induce effective DC maturation, leading to the induction of humoral and cellular immunities to sufficient levels for protecting viral infections. Further studies on the regulation of intracellular trafficking of this nanocarrier are necessary for optimizing the ratio of humoral immunity to cellular immunity of APC-targeting BNC-based vaccines, which is indispensable for developing best vaccine against each pathogen.

## Methods

Materials used in this study are described in Additional file 1.

### Preparation of OVA-conjugated α-DC-ZZ-BNC

OVA was conjugated with ZZ-BNC by using two types of chemical crosslinker (Sulfo-LC-SPDP and SPDP; Pierce) as described previously [23]. Either anti-CD11c IgG or Armenian hamster IgG isotype control was displayed onto ZZ-BNC-OVA as described previously (Additional file 1) [33]. Z-averages and ζ-potentials of ZZ-BNCs were measured in water at 25 °C by a dynamic light scattering model Zetasizer Nano ZS (Malvern Instruments, Worcestershire, UK).

### Ex vivo cellular uptake analysis of splenic DCs

The care of the animals and all procedures used in these experiments were approved by the Committee on Animal Experiments of Graduate School of Bioagricultural Sciences, Nagoya University. Spleens were isolated from C57BL/6 mice (6 weeks, female, Japan SLC, Inc., Hamamatsu, Japan), and splenocytes were prepared (Additional file 1). Splenic DCs (approximately 2.0 × 10^5^ cells) were mixed with CF488A-labeled α-DC-ZZ-BNC-OVA (2 μg as OVA), incubated in RPMI1640 medium supplemented with 10% (v/v) FBS at 37 °C for 4 h. The cells were washed and analyzed by a flow cytometer BD FACS Canto II (BD Biosciences, San Jose, CA, USA). Splenic DCs loaded with CF488A-labeled α-DC-ZZ-BNC-OVA were stained with Wheat Germ Agglutinin Alexa Fluor 633 Conjugate (Life Technologies). The cells were observed under a confocal laser scanning microscopy (LSM) model FV-1000D (Olympus, Tokyo, Japan). Whole cell Z-stacks (each slice = 0.5 μm, total 14 sections) were acquired by LSM, which was equipped with a × 100 oil objective lens (Olympus).

### In vitro cellular uptake analysis of DC2.4 cells

Approximately 2 × 10^4^ of DC2.4 cells (a mouse bone marrow DC-derived cell line) cultured in an 8-well glass-bottomed chamber were contacted with the α-DC-ZZ-BNC-fOVA complexes (1.2 μg as fOVA) for 3 h, and incubated in fresh medium for 23 h. Following the treatment with 50 nM of LysoTracker Red (Life Technologies) for 1 h, the cells were washed twice with PBS, fixed in 4% (w/v) PFA in PBS for 20 min. The fluorescence was observed under LSM.

### In vivo distribution analysis

CF750-labeled α-DC-ZZ-BNC (10 μg as ZZ-L protein) was injected to Balb/c mice (6–8 weeks, female; Japan SLC, Inc.) through SC or IM routes. After 12 h, the mice were sacrificed, and tissues were isolated. CF750-derived fluorescence in each tissue was observed by using an in vivo imaging system OV-100 (Olympus), and then semi-quantified with an image processing program FV10-ASW version 4 (Olympus).

### In vivo accumulation analysis of APCs in LNs

CF633-labeled α-DC-ZZ-BNC (10 μg as ZZ-L protein) was injected to Balb/c mice through SC or IM routes. After 12 h, the mice were sacrificed and then subjected to the isolation of LNs closest to injection sites. Following homogenization with ground glasses, the released cells were stained with α-CD11c-FITC, α-CD11b-FITC, and α-CD19-FITC (Miltenyi Biotech). These cells were analyzed by using a flow cytometer.

### Ex vivo DC maturation analysis

Splenic DCs (approximately 5.0 × 10^5^ cells) grown on a 24-well plate were incubated with 10 μg (as OVA) of OVA, ZZ-BNC-OVA, or α-DC-ZZ-BNC-OVA at 37 °C for 24 h. Lipopolysaccharides (LPS) (10 μg; Sigma Aldrich) were used as a positive control. After 24 h incubation, the cells were collected, and stained with APC-labeled anti-CD80 IgG (Miltenyi Biotech), anti-CD86 IgG (Miltenyi Biotech), or anti-CD40 IgG (Miltenyi Biotech). The APC-derived fluorescence was analyzed by using a flow cytometer.

### In vivo OVA-specific T cell responses analysis

C57BL/6 mice were injected with α-DC-ZZ-BNC-OVA (10 μg as OVA) subcutaneously. After 6 days, splenocytes were isolated and stained with APC-labeled H-2k^b^ OVA tetramer-SIINFEKL (MBL, Nagoya, Japan) and FITC-labeled anti-CD8 IgG (MBL) for detection of OVA-specific CD8^+^ T lymphocytes, and then subjected to flow cytometry.

### In vivo OVA-specific IgG production analysis

C57BL/6 mice (female, 6 weeks) were injected with α-DC-ZZ-BNC-OVA (10 μg as OVA) subcutaneously at week 0, 2, and 4. Blood samples (about 50 μl) were collected from the tail vein at week 6. Titers of OVA-specific IgGs (IgG1 and IgG2a) in sera were determined by using ELISA (Additional file 1).

### JEV protection assay

JEV envelope-derived D3 protein was prepared as described previously [34]. According to the preparation protocol of α-DC-ZZ-BNC-OVA, SPDP-treated D3 protein was conjugated with sulfo-LC-SPDP-treated ZZ-BNC, and the ZZ-BNC-D3 complex was modified with anti-CD11c IgG to form α-DC-ZZ-BNC-D3 complex. As a control vaccine, D3 protein was conjugated with α-CD11c IgG (α-CD11c-D3).

Balb/c mice (7 weeks, female) were injected with α-DC-ZZ-BNC-D3 (30 μg as D3 protein) subcutaneously at weeks 0, 2, and 4, and then subjected to blood collection from tail vein at week 6. As published elsewhere [34], the mice were challenged intraperitoneally with 50-folds higher dose of 50% lethal dose of the JEV JaGAr01 strain followed by intracerebral inoculation with PBS. The mice were subjected to monitoring the signs of illness/distress such as ruffled fur or paralysis and recording the survival rates. Titers of anti-JEV IgGs (including anti-D3 IgGs) in sera were determined by using ELISA as described previously [34].

## Additional file

**Additional file 1.** Additional Methods includes information of materials and detailed methods.
