# Supplementary material for: CD11c-specific bio-nanocapsule enhances vaccine immunogenicity by targeting immune cells
Source: J Nanobiotechnology. 2018 Aug 4;16:59. doi: 10.1186/s12951-018-0386-6 (PMC6076409; doi:10.1186/s12951-018-0386-6)
Supplement: Supplementary file 1 — Additional file 1. Additional Methods includes information of materials and detailed methods. [file 12951_2018_386_MOESM1_ESM.docx]

Additional Methods

*Materials*

Endotoxin-free OVA was purchased from Invivogen (Endofit OVA; San Diego, CA, USA). ZZ-BNCs were overexpressed in *Saccharomyces cerevisiae* AH22R^-^ cells carrying ZZ-BNC-expression plasmid pGLD-ZZ50 ^1^, and purified as described previously ^2,3^. Protein concentrations were determined with a micro BCA protein assay kit (Pierce, Rockford, IL, USA) using bovine serum albumin as a standard protein. OVA was labeled with a CF488A Maleimide (Biotium, Heyward, CA, USA), according to the manufacturer’s protocol, which was designated fOVA. BNC was also labeled with a CF633 Succinimidyl Ester or a CF750 Succinimidyl Ester (Biotium). Fluorophore-labeled 40-nm polystyrene beads (FluoSphere; carboxylate-modified, yellow-green) were purchased from Life Technologies (Carlsbad, CA, USA). Antibodies for the DC targeting are as follows; Armenian hamster monoclonal anti-CD11c IgG (clone N418; eBioscience, San Diego, CA, USA) and Armenian hamster IgG isotype control (clone eBio299Arm; eBioscience). DC2.4 cells were kindly provided by Dr. K. L. Rock (Harvard Medical School, Boston, MA, USA).

*Conjugation of anti-CD11c IgG on ZZ-BNC*

Anti-CD11c IgG (1 μg) was mixed with ZZ-BNC-OVA (5 μg as ZZ-L protein) in the presence of 50 μM bis-sulfosuccinimidyl suberate (BS^3^, Pierce), and then incubated at room temperature for 1 h. Crosslinking reaction was stopped by the addition of glycine-NaOH (pH 7.5, 100 μM, final concentration). The anti-CD11c IgG-displayed ZZ-BNC-OVA was designated as α-DC-ZZ-BNC-OVA.

*Isolation of splenic DCs*

Spleens were isolated from C57BL/6 mice (6 weeks, female, Japan SLC, Inc., Hamamatsu, Japan), and splenocytes were prepared with gentleMACS dissociator (Miltenyi Biotech, Bergisch Gladbach, Germany) in the presence of 2 mg/ml collagenase D (Roche, Mannheim, Germany). CD11c^+^ cells were purified from the splenocytes by a magnetic-activated cell sorting (MACS, Miltenyi Biotech) using an FcR Blocking Reagent and an α-CD11c IgG (clone N418)-conjugated magnetic beads. The cell preparations containing >80% CD11c^+^ cells (viability, >95%) were used as splenic DCs.

*ELISA*

For the measurement of OVA-specific IgG production analysis, 96-well plates were coated with 50 μl of 20 μg/ml OVA in bicarbonate buffer (pH 9.6) at 4 ˚C for overnight, and blocked with 10% (w/v) skimmed milk (Wako, Osaka, Japan) at 37 ˚C for 2 h. Serially diluted sera (50 μl) were added to each well and incubated at 37 ˚C for 2 h. After washing with PBST (PBS containing 0.1% (v/v) Tween 20) three times and PBS three times, horseradish peroxidase (HRP)-conjugated form (200 ng/ml) of anti-mouse IgG1 antibody (eBioscience) or anti-mouse IgG2a antibody (Abcam, Cambridge, UK) was added to each well, and incubated at 37 ˚C for 2 h. After washing with PBST three times and PBS three times, 100 μl of 3,3’-5,5’-tetramethylbenzidine (TMB) substrate (Pierce) was added to each well, and incubated at room temperature for 20 min. The reaction was stopped by adding 50 μl of 2 N sulfuric acid, and the absorbance was measured at 450 nm (OD_450_). Based on the OD_450_ value of 100-fold diluted non-immune sera (n = 4), the cut-off value (COV) was defined as the mean plus 2-fold standard deviation (SD) of OD_450_ values; COV = [mean + (SD × 2)]. Titers of anti-OVA IgGs were defined as the highest serum dilution factor at which the OD_450_ value became closest above COV.

For the measurement of JEV-specific IgG, 96-well plates were coated with 50% (v/v) Japanese Encephalitis TC Vaccine (Kaketsuken, Kumamoto, Japan) in bicarbonate buffer (pH 9.6) at 4 ˚C for overnight. According to the ELISA protocol for OVA-specific IgGs (see above), anti-JEV IgGs in sera were determined with HRP-conjugated anti-mouse total IgG secondary antibody (Sigma Aldrich) and TMB substrate. After measuring OD_450_ values, titers were calculated by the same method for OVA-specific IgGs.

1. Tsutsui Y, Tomizawa K, Nagita M, Michiue H, Nishiki TI, Ohmori I, Seno M, Matsui H. Development of bionanocapsules targeting brain tumors. *J. Control. Release*. 2007;**122**(2), 159–164. doi:10.1016/j.jconrel.2007.06.019.

2. Jung J, Iijima M, Yoshimoto N, Sasaki M, Niimi T, Tatematsu K, Jeong S-YY, Choi EK, Tanizawa K, Kuroda S. Efficient and rapid purification of drug- and gene-carrying bio-nanocapsules, hepatitis B virus surface antigen L particles, from Saccharomyces cerevisiae. *Protein Expr. Purif.* 2011;**78**(2), 149–55. doi:10.1016/j.pep.2011.04.008.

3. Iijima M, Matsuzaki T, Kadoya H, Hatahira S, Hiramatsu S, Jung G, Tanizawa K, Kuroda S. Bionanocapsule-based enzyme-antibody conjugates for enzyme-linked immunosorbent assay. *Anal. Biochem.* 2010;**396**(2), 257–61. doi:10.1016/j.ab.2009.10.010.
